# Loss of 4E-BP1 function induces EMT and promotes cancer cell migration and invasion via cap-dependent translational activation of snail

**DOI:** 10.18632/oncotarget.2109

**Published:** 2014-06-16

**Authors:** Weijia Cai, Qing Ye, Qing-Bai She

**Affiliations:** ^1^ Markey Cancer Center, University of Kentucky College of Medicine, Lexington, KY, USA; ^2^ Department of Molecular and Biomedical Pharmacology, University of Kentucky College of Medicine, Lexington, KY, USA

**Keywords:** 4E-BP1, mTORC1, Snail, EMT, migration, invasion

## Abstract

The cap-dependent translation is frequently deregulated in a variety of cancers associated with tumor progression. However, the molecular basis of the translation activation for metastatic progression of cancer remains largely elusive. Here, we demonstrate that activation of cap-dependent translation by silencing the translational repressor 4E-BP1 causes cancer epithelial cells to undergo epithelial-mesenchymal transition (EMT), which is associated with selective upregulation of the EMT inducer Snail followed by repression of E-cadherin expression and promotion of cell migratory and invasive capabilities as well as metastasis. Conversely, inhibition of cap-dependent translation by a dominant active mutant 4E-BP1 effectively downregulates Snail expression and suppresses cell migration and invasion. Furthermore, dephosphorylation of 4E-BP1 by mTORC1 inhibition or directly targeting the translation initiation also profoundly attenuates Snail expression and cell motility, whereas knockdown of 4E-BP1 or overexpression of Snail significantly rescues the inhibitory effects. Importantly, 4E-BP1-regulated Snail expression is not associated with its changes in the level of transcription or protein stability. Together, these findings indicate a novel role of 4E-BP1 in the regulation of EMT and cell motility through translational control of Snail expression and activity, and suggest that targeting cap-dependent translation may provide a promising approach for blocking Snail-mediated metastatic potential of cancer.

## INTRODUCTION

The cap-dependent translation is a process by which most capped mRNAs are translated into proteins. This process is limited by translation initiation, a step controlled by the eIF4F complex at the level of ribosomal recruitment. The eIF4F complex is assembled by the translation initiation factor eIF-4E that binds to the 5′-cap structure of mRNAs, the scaffolding protein eIF4G and the RNA helicase eIF4A [1]. Emerging evidence indicates that translation of certain key oncogenic mRNAs bearing long and highly structured 5′-untranslated regions (UTR), such as those encoding proliferation- and survival-promoting proteins including cyclins, VEGF, c-Myc, Bcl-2 and survivin, is strongly dependent on the eIF-4E. Consequently, these oncogenic mRNAs are selectively and disproportionately affected by eIF4E availability and are sensitive to the alteration in the levels of eIF4E [2, 3]. Overexpression of eIF4E is frequently observed in a variety of human cancers. In addition, another major mechanism for regulation of eIF4E function is involved in the eIF4E-binding proteins, 4E-BPs. 4E-BP1 (the major epithelial cell form) is a member of the 4E-BP family that represses translation by competing with eIF4G for binding to eIF4E, thereby preventing formation of the eIF4F complex. In cancer cells, however, 4E-BP1 is frequently hyperphosphorylated by its upstream oncogenic signals such as PI3K/AKT and RAS/RAF/MEK/ERK pathways, which causes 4E-BP1 disassociation from eIF4E and thus inactivates 4E-BP1 function and increases the level of free eIF4E. The mTOR kinase complex 1 (mTORC1), a downstream target of both AKT and ERK signaling [4, 5], is a master regulator of eIF4E-initiated cap-dependent translation by phosphorylating 4E-BP1 on Thr37 and Thr46 that act as priming sites for its subsequent phosphorylation on Ser65 and Thr70 [6]. Hyperphosphorylation of 4E-BP1 or reduction of 4E-BP1 expression is associated with malignant progression and poor prognosis [3, 7-9], whereas dephosphorylation of 4E-BP1 has been identified as an important biomarker for predicting response to anti-cancer therapy [10, 11]. These findings have indicated that deregulation of 4E-BP1-controlled cap-dependent translation can contribute to cancer progression and support the development of agents that target 4E-BP1 phosphorylation or eIF-4E.

Snail (Snail1) is a zinc-finger transcription factor that functions as a key transcriptional repressor in inhibiting E-cadherin expression [12, 13]. E-cadherin is a transmembrane glycoprotein that mediates epithelial intercellular junctions. Downregulation of E-cadherin is a hallmark of epithelial-mesenchymal transition (EMT) during embryonic development, a process also exploited by invasive cancer cells [14, 15]. During EMT, epithelial tumor cells lose their epithelial polarity and cell-cell adhesion and gain mesenchymal phenotypes with increased migratory and invasive capabilities. It has long been believed that EMT is initiated at the advanced stage of tumor progression and is a prerequisite for tumor cell dissemination and metastasis [14, 15]. Numerous experimental systems showed that Snail represses E-cadherin expression and induces EMT in different type of cancer cells, indicating that Snail plays a fundamental role in EMT and cancer metastasis [14, 15]. Indeed, there is a considerable inverse correlation between Snail and E-cadherin expression in a variety of epithelial tumors. Overexpression of Snail or reduced E-cadherin expression correlates with higher tumor grade, nodal metastasis and tumor recurrence, and predicts poor clinical outcomes in patients with various cancers [12-15]. Snail expression is induced by a wide range of signaling pathways including TGFβ, Notch and Wnt pathways and hypoxia stress [16]. Phosphorylation of Snail by GSK3β and PAK1 plays an important role in regulation of its stability, cellular localization and function [17, 18]. However, little is known about whether Snail is regulated at the level of translation.

In the present study, we demonstrate that activation of cap-dependent translation by loss of 4E-BP1 function in cancer cells selectively upregulates Snail expression and enhances its functions on EMT, cell migration/invasion and metastasis. Our study uncovers an important aspect of translational regulation of Snail in controlling cell motility and metastatic potential of cancer.

## RESULTS

### Reduction of 4E-BP1 expression induces EMT, upregulates Snail expression and promotes cancer cell migration, invasion and metastasis

**Figure 1:**
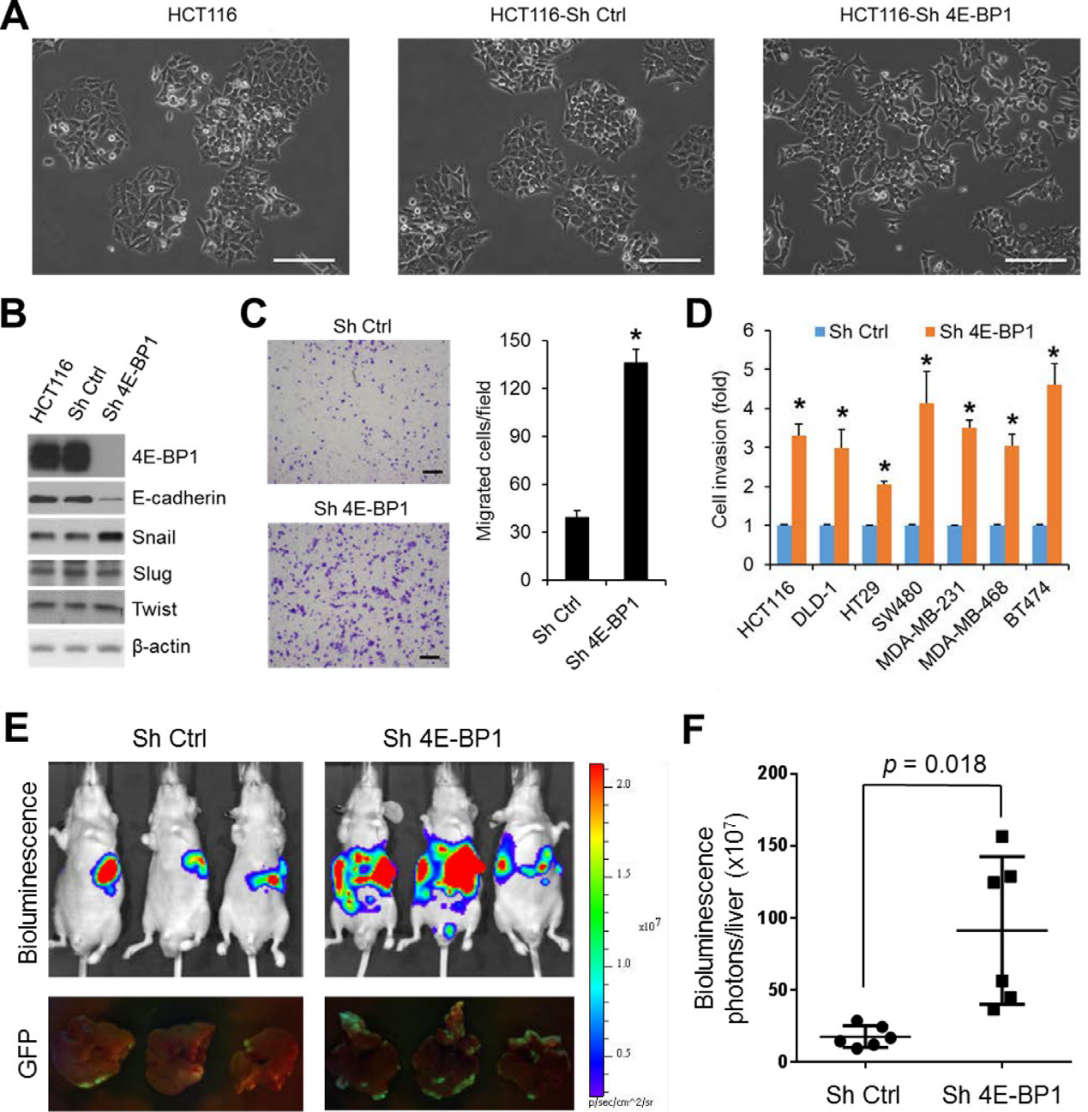
Silencing of 4E-BP1 induces EMT, upregulates Snail expression, and enhances cancer cell migration, invasion and metastasis (A) For morphological comparison, HCT116 cells alone or with stable expression of control (Ctrl) shRNA or 4E-BP1 shRNA cells were photographed using a light microscope. Scale bar = 200 μm. (B) HCT116 cells alone or with stable expression of Ctrl shRNA or 4E-BP1 shRNA were immunoblotted with the indicated antibodies. (C) Transwell migration analysis of HCT116 cells with stable expression of Ctrl shRNA or 4E-BP1 shRNA over 6 h. The results represent the mean number of migrated cells per field ± S.E.M. (n=3). Scale bar = 500 μm. (D) The invasive ability of various cancer cell lines with stable expression of Ctrl shRNA or 4E-BP1 shRNA. The results are expressed as the fold change of cell invasion in Sh 4E-BP1 cells relative to the Sh Ctrl cells and presented as means ± S.E.M. (n=3). (E) Bioluminescence and GFP images of liver metastasis in athymic nude mice injected intrasplenically with HCT116-Luc/GFP cells expressing Ctrl shRNA or 4E-BP1 shRNA at week 3 post-injection. (F) Quantitative analysis of bioluminescence in liver metastasis as shown in (E) (n=6 mice/group). * *P* < 0.02 for Sh 4E-BP1 versus Sh Ctrl.

4E-BP1 expression has been shown to be inversely correlated with tumor progression in colorectal, breast and other cancers [8-10]. To investigate the functional importance and molecular basis of 4E-BP1-regulated cap-dependent translation in cancer progression, we generated HCT116 colon cancer cell derivatives with stable knockdown of 4E-BP1 expression by shRNA. As compared to the morphology of HCT116 cells or HCT116 cells expressing control shRNA under a light microscope, we surprisingly observed that knockdown of 4E-BP1 expression caused HCT116 cells acquired fibroblastic-like characteristics, as they demonstrated a more elongated morphology and a scattered density, whereas the isogenic controls or parental cells exhibited higher degree of adherence between neighboring cells (Figure 1A). Similar results were also observed in silencing 4E-BP1 expression in DLD1 colon cancer cells (Supplementary Figure 1A). The observed alterations are characteristic features of EMT. The loss of E-cadherin is considered to be the most fundamental event during EMT [14, 15]. Intriguingly, we found that cells (HCT116, DLD1, SW480, BT474) with stable knockdown of 4E-BP1 expression demonstrated a marked reduction in the level of E-cadherin protein (Figure 1B and Supplementary Figure 1B). To understand how E-cadherin was downregulated by 4E-BP1 knockdown, we determined the expression of several key transcriptional factors known to facilitate EMT by repression of E-cadherin. Remarkably, the expression of Snail, a direct transcriptional repressor of E-cadherin, was dramatically upregulated in those cells with 4E-BP1 knockdown (Figure 1B and Supplementary Figure 1B). In contrast, expression of other E-cadherin transcriptional repressors such as Slug (Snail2) and Twist was unaffected. Emerging evidence indicates that induction of EMT by Snail contributes cells granted with motility and invasion capacities [14, 15]. We thus examined the effect of 4E-BP1 knockdown on cell migration and invasion using Boyden chamber assays as we described previously [19]. As shown in Figure 1C, D, HCT116 cells with 4E-BP1 knockdown exhibited a three- to five-fold increase in both migratory and invasive capacities compared with the control cells. Similar results were also obtained in three other colon cancer cell lines (DLD1, HT29, SW480) and three breast cancer cell lines (MDA-MB-231, MDA-MB-468, BT474) in which 4E-BP1 gene was silenced (Figure 1D, migration data not shown). To determine whether the increased migratory and invasive abilities of cancer cells by 4E-BP1 knockdown facilitate cancer metastasis, we used an experimental liver metastasis model of colon cancer *in vivo* as we described previously [19]. Luciferase and GFP-labeled HCT116 cells with stable 4E-BP1 knockdown were injected intrasplenically into athymic nude mice. Formation of liver metastasis was assessed by bioluminescent and fluorescent imaging. Compared to the HCT116 cells expressing control shRNA, silencing 4E-BP1 expression markedly promoted liver metastases in mice (Figure 1E, F). Collectively, these results suggest that 4E-BP1 loss selectively upregulates Snail protein expression for EMT induction and subsequently enhances cancer cell migration and invasion as well as metastasis.

### Dephosphorylated 4E-BP1 inhibits Snail expression and cancer cell migration and invasion

**Figure 2:**
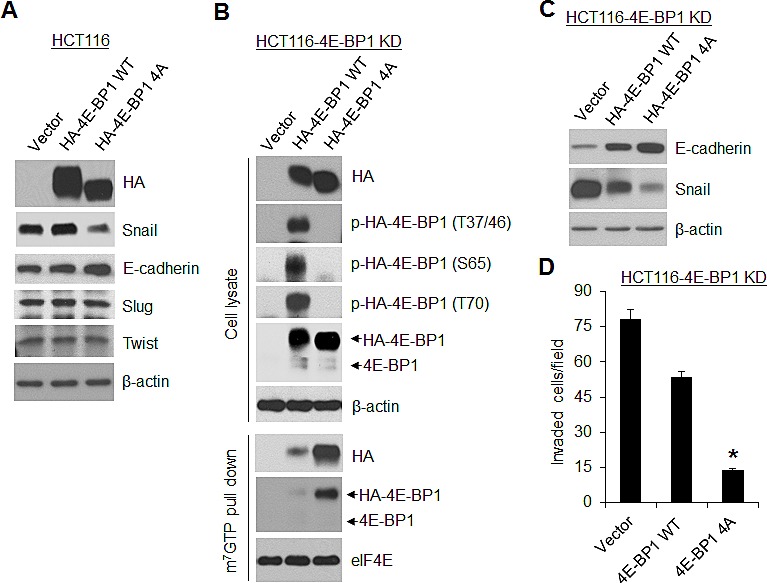
A dominant active 4E-BP1 mutant profoundly inhibits Snail expression and cell invasion (A) Immunoblot analysis of HCT116 cells with stable expression of vector, HA-4E-BP1 WT or HA-4E-BP1 4A. (B, C) HCT116-4E-BP1 knockdown (KD) cells were re-expressed with vector, HA-4E-BP1 WT or HA-4E-BP1 4A. Cell lysates were immunoblotted with the indicated antibodies or precipitated with m^7^GTP sepharose beads followed by immunoblotting of HA, 4E-BP1 and eIF4E. (D) Transwell invasion analysis of HCT116-4EBP1 KD cells with re-expressing vector, HA-4E-BP1 WT or HA-4E-BP1 4A over 30 h. The results represent the mean number of invaded cells per field ± S.E.M. (n=3). * *P* < 0.02 for 4E-BP1 4A versus 4E-BP1 WT or vector.

Loss of 4E-BP1 expression or hyperphosphorylation of 4E-BP1 is known to lead to activation of cap-dependent translation [1]. To ascertain the role of cap-dependent translation in the regulation of Snail expression and cell migration and invasion, 4E-BP1 wild-type (wt) and its mutant 4E-BP1-4A, in which the four known phosphorylation sites (T37, T46, S65, T70) were replaced with alanine were ectopically expressed in HCT116 cells. We showed previously that the mutant 4E-BP1-4A cannot be phosphorylated and binds constitutively to eIF4E, thus inhibits cap-dependent translation, whereas expression of 4E-BP1 wt had no such effects due to its hyperphosphorylation in HCT116 cells [11]. As compared to 4E-BP1 wt and vector control, expression of the dominant active 4E-BP1-4A mutant profoundly repressed expression of Snail but not Slug and Twist (Figure 2A), and additionally inhibited cell migration and invasion as we showed previously [19]. Similar results were also obtained in MDA-157 breast cancer cells by expression of the active 4E-BP1-4A mutant (Supplementary Figure 2). To further confirm the role of 4E-BP1 in regulation of Snail activity, 4E-BP1 wt and 4A were re-expressed in HCT116-4E-BP1 knockdown cells. Consistent with our previous findings [11] and the results as indicated above, expressed 4E-BP1-4A bound constitutively to eIF4E-mRNA cap complex and markedly inhibited Snail expression attendant with a dramatic increase in the level of E-cadherin and suppression of cell invasion (Figure 2B, C and D). In contrast, 4E-BP1 wt was highly phosphorylated at the four phosphorylation sites; only slightly bound to eIF4E-mRNA cap complex; and thus had much less inhibitory effect on Snail expression and cell invasion than those induced by 4E-BP1-4A. These data suggest that the phosphorylation status of 4E-BP1 is associated with its function on the regulation of Snail expression and its activity.

**Figure 3:**
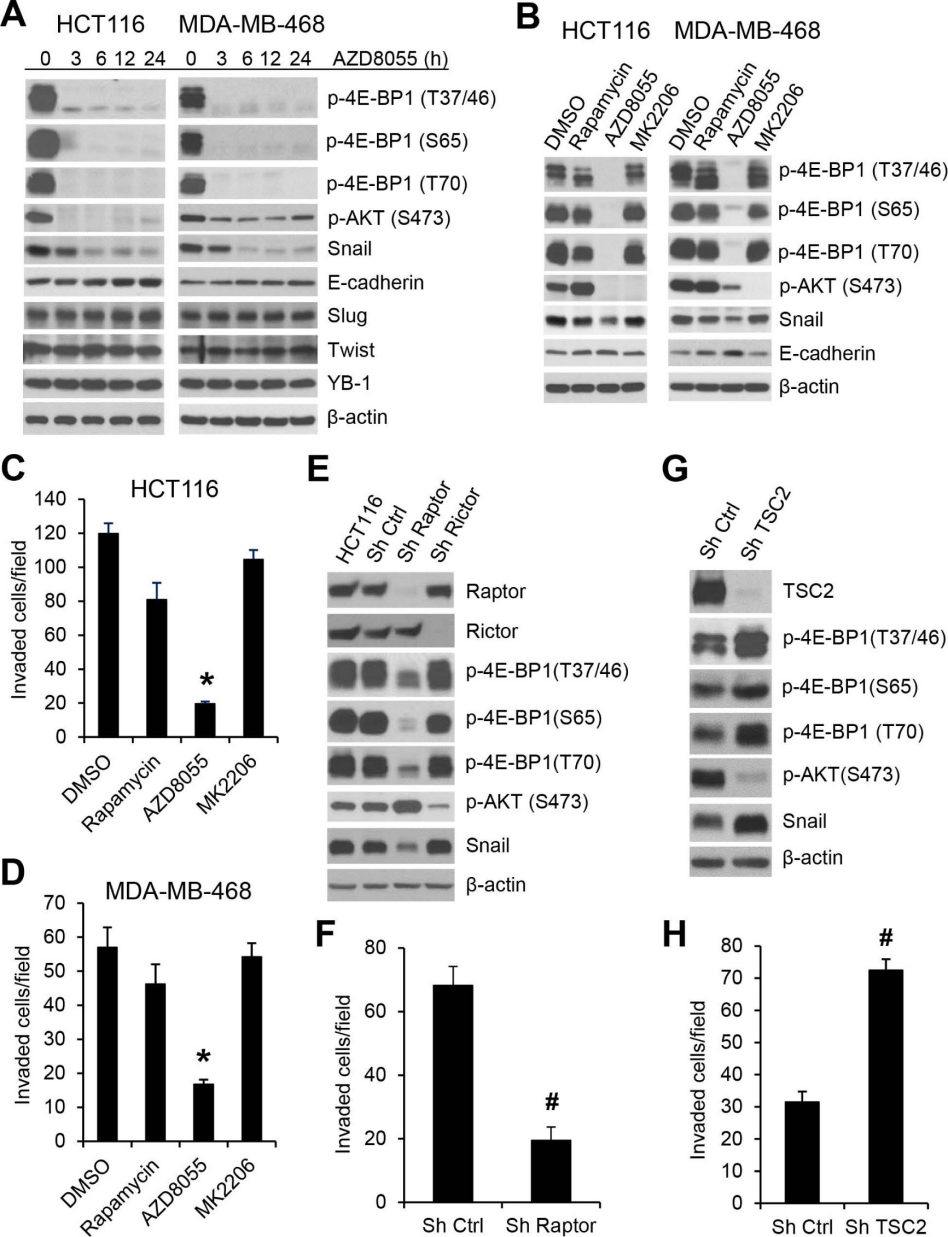
mTORC1 plays a critical role in the regulation of Snail expression and cancer cell motility (A) HCT116 or MDA-MB-468 cells were treated with 500 nM AZD8055 for the indicated times. Cell lysates were immunoblotted with the indicated antibodies. (B) HCT116 or MDA-MB-468 cells were treated with 50 nM rapamycin. 500 nM AZD8055, 1 μM MK2206 or dimethyl sulfoxide (DMSO) as control for 12 h. Cell lysates were immunoblotted with the indicated antibodies. (C, D) Transwell invasion analysis of HCT116 and MDA-MB-468 cells in the presence of the drugs as indicated in (B) for 30 h (C) and 24 h (D), respectively. The results represent the mean number of invaded cells per field ± S.E.M. (n=3). * *P* < 0.03 for AZD8055 versus DMSO control, rapamycin or MK2206. (E) HCT116 cells alone or with stable expression of Ctrl shRNA, raptor shRNA or rictor shRNA were immunoblotted with the indicated antibodies. (F) Transwell invasion analysis of HCT116 cells with stable expression of Ctrl shRNA or raptor shRNA over 30 h. (G) Immunoblot analysis of HCT116 cells with stable expression of Ctrl shRNA or TSC2 shRNA. (H) Transwell invasion analysis of HCT116 cells with stable expression of Ctrl shRNA or TSC2 shRNA over 30 h. The results (F, H) represent the mean number of invaded cells per field ± S.E.M. (n=3). ^#^ *P* < 0.03 for Sh raptor or Sh TSC2 versus Sh Ctrl.

The mTOR kinase forms two distinct functional complexes, mTORC1 and mTORC2. mTORC1 is a master regulator of cap-dependent translation by phosphorylation of 4E-BP1, whereas mTORC2 regulates AKT activity through phosphorylation of AKT on Ser473 [20]. Rapamycin is a modest inhibitor of mTORC1 activity and mTOR kinase inhibitors are much more effective than rapamycin in inhibiting 4E-BP1 phosphorylation [21, 22]. Using a clinical-grade ATP-site mTOR kinase inhibitor AZD8055 [23], we explored whether mTORC1 inhibition also suppresses Snail expression and cancer cell migration and invasion. As shown in Figure 3A, B and Supplementary Figure 3, AZD8055 effectively inhibited phosphorylation of 4E-BP1 at the four phosphorylation sites in the three tested cell lines (HCT116, MDA-MB-468 and MDA-MB-231). Inhibition of 4E-BP1 phosphorylation in these cells was associated with downregulation of Snail expression accompanied by an increase in E-cadherin expression upon AZD8055 exposure within 24 h (Figure 3A). In contrast, AZD8055 had no effect on the expression of other E-cadherin regulators, Slug and Twist, or a recently reported Snail regulator, Y-box binding protein 1 (YB-1) [24]. As compared to AZD8055, rapamycin, an allosteric inhibitor of mTORC1, only weakly inhibited 4E-BP1 phosphorylation and had much less repression on Snail expression and cell invasion (Figure 3B, C and D). Since AZD8055 also inhibited mTORC2 as indicated by loss of p-AKT on S473 (Figure 3A, B), we investigated whether inhibition of AKT by AZD8055 is associated with reduction of Snail expression. Treatment with MK2206, a selective AKT inhibitor [25], effectively inhibited AKT phosphorylation but did not affect 4E-BP1 phosphorylation, Snail expression and cell invasion in HCT116 and MDA-468 cells (Figure 3B, C and D). Furthermore, knockdown of rictor, a key component of mTORC2 [26], inhibited p-AKT but had no effect on Snail expression (Figure 3E). However, depletion of raptor, an obligatory component of mTORC1 [27], markedly repressed 4E-BP1 phosphorylation, Snail expression and cell invasion (Figure 3E, F). Conversely, activation of mTORC1 by silencing its upstream repressor TSC2 [28] enhanced 4E-BP1 phosphorylation, Snail expression and cell invasion (Figure 3G, H), although mTROC1 activation by TSC2 knockdown induced feedback inhibition of AKT that was consistent with the previous reports [29, 30]. Taken together, these results indicate that the mTORC1/4E-BP1 signaling axis plays an indispensable role in the regulation of Snail expression and migratory/invasive capacities of cancer cells.

### mTORC1/4E-BP1 signaling regulates Snail expression and its activity at the level of translation

**Figure 4:**
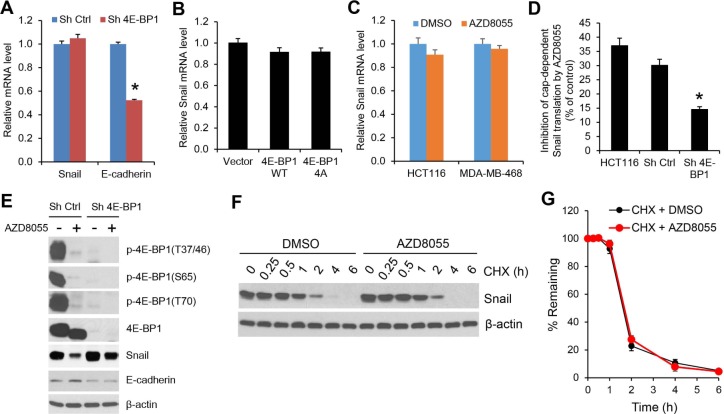
The regulation of Snail expression by mTORC1 is through 4E-BP1-mediated cap-dependent translation mechanism (A) Quantitative RT-PCR analysis of mRNA expression of Snail or E-cadherin relative to β-actin in HCT116 cells with stable expression of Ctrl shRNA or 4E-BP1 shRNA (n=3). (B) Quantitative RT-PCR analysis of mRNA expression of Snail relative to β-actin in HCT116 cells with stable expression of vector, HA-4E-BP1 WT or HA-4E-BP1 4A (n=3). (C) Quantitative RT-PCR analysis of mRNA expression of Snail relative to β-actin in HCT116 cells that were treated with 500 nM AZD8055 or DMSO as control for 24 h (n=3). (D) HCT116 cells alone or with stable expression of Ctrl shRNA or 4E-BP1 shRNA were transfected with a bicistronic luciferase reporter that detects cap-dependent translation of the *Renilla* luciferase gene linked to the 5′-UTR of Snail and cap-independent Polio IRES-mediated translation of the firefly luciferase gene. The transfected cells were treated with 500 nM AZD8055 or DMSO as control for 12 h. Luciferase activities were measured by a dual-luciferase assay, and the *Renilla*/firefly luciferase luminescence ratio was calculated for cap-dependent translational activity. The results are expressed as the inhibition of cap-dependent translation relative to the DMSO-treated controls and presented as means ± S.E.M. (n=3). (E) Immunoblot analysis of HCT116 cells with stable expression of Ctrl shRNA or 4E-BP1 shRNA that were treated with or without 500 nM AZD8055 for 12 h. (F) HCT116 cells were treated with 500 nM AZD8055 or DMSO as control for 30 min, followed by addition of 20 μg/ml cycloheximide (CHX) for the indicated times. Cell lysates were immunoblotted with Snail and β-actin antibodies. (G) Immunoblots of Snail as shown in (F) were quantified using the FluorChem digital imaging system (Alpha Innotech, Santa Clara, CA). The level of Snail remaining was obtained by normalizing β-actin level at each time, and the results are presented as mean ± S.E.M. (n=3). * *P* < 0.03 for Sh 4E-BP1 versus Sh Ctrl.

To understand how mTORC1/4E-BP1 signaling regulates Snail expression, we first determined the level of Snail mRNA by quantitative real-time reverse transcription-PCR (RT-PCR). As compared to the upregulation of Snail protein expression elicited by 4E-BP1 knockdown in HCT116 cells (Figure 1B), there was not a significant change in the level of Snail mRNA between 4E-BP1 knockdown and control cells (Figure 4A). However, the level of E-cadherin mRNA in the 4E-BP1 knockdown cells was inhibited by about 50% (Figure 4A), which was associated with its decreased protein expression (Figure 1B). These data suggest that 4E-BP1 translationally regulates Snail expression and secondarily affects its transcriptional targets such as E-cadeherin. This notion was further supported by the results showing that downregulation of Snail protein by inhibition of cap-dependent translation with 4E-BP1 4A (Figure 2A) or the mTOR kinase inhibitor AZD8055 (Figure 3A, B) was not associated with its change in the level of mRNA (Figure 4B, C). In addition, using a cap-dependent translation reporter luciferase (Luc) mRNA linked to the 5′-UTR of Snail for measurement of cap-dependent Snail translation activity, we found that the 5′-UTR of Snail-Luc translation activity was inhibited (30-37%) by AZD8055 in HCT116 cells or HCT116 cells expressing control shRNA, but silencing 4E-BP1 expression that activates cap-dependent translation significantly rescued the inhibitory effect (Figure 4D). Moreover, knockdown of 4E-BP1 expression markedly reversed the decreased expression of Snail protein attendant with the increased expression of E-cadherin, and reduced the inhibitory effect of AZD8055 on cell invasion (Figure 4E and Supplementary Figure 4). Additionally, we found that the decreased Snail expression by AZD8055 was not due to the change in its protein stability as analyzed by protein degradation rate using cycloheximide chase assay (Figure 4F, G). Collectively, these data indicate that mTORC1 regulates Snail expression and its activity largely through 4E-BP1-mediated cap-dependent translation mechanism.

### Snail mediates the effects of mTORC1/4E-BP1 signaling on translational control of cancer cell migration and invasion

**Figure 5:**
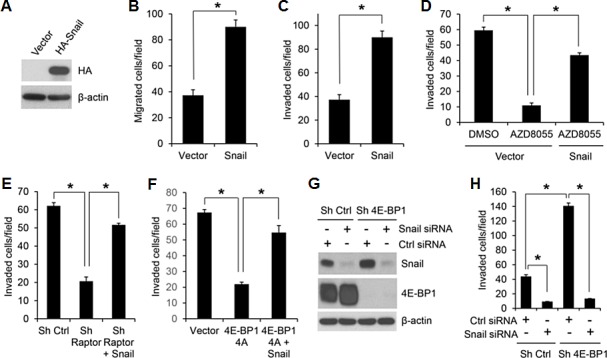
Snail is an important effector of mTORC1/4E-BP1 signaling responsible for translational control of cancer cell migration and invasion (A) Immunoblot analysis of HCT116 cells with expression of vector or HA-tagged Snail. (B, C) Migration (B) or invasion (C) analysis of HCT116 cells with expression of vector or Snail. (D) Invasion analysis of HCT116 cells with expression of vector or Snail in the presence of 500 nM AZD8055 or DMSO as control for 30 h. (E) Invasion analysis of HCT116 cells with stable expression of control shRNA, raptor shRNA or raptor shRNA with co-expressing Snail over 30 h. (F) Invasion analysis of HCT116 cells with stable expression of vector, 4E-BP1 4A or 4E-BP1 4A with co-expressing Snail over 30 h. (G, H) HCT116 cells with stable expression of Ctrl shRNA or 4E-BP1 shRNA were transfected with control siRNA or Snail siRNA for 48 hours, followed by immunoblot analysis with the indicated antibodies (G) and invasion analysis over 30 h (H). Data shown in graphs represent the mean ± S.E.M. (n=3). * *P* < 0.02.

The importance of Snail downregulation in mediating the effects of the mTORC1/4E-BP1 pathway inhibition was determined in HCT116 cells in which Snail protein was exogenously overexpressed (Figure 5A). These cells did not exhibit changes in cell proliferation (data not shown) but showed a two- to three-fold increase in cell migration and invasion compared with the vector control cells (Figure 5B, C). Furthermore, in these cells, the effect of mTROC1 inhibition elicited by AZD8055 or raptor knockdown on suppression of cell invasion was profoundly reduced compared with that in the control cells (Figure 5D, E). In addition, overexpression of Snail could also reverse a decreased cell invasion induced by the cap-dependent translation inhibitor 4E-BP1 4A (Figure 5F). Conversely, silencing Snail expression 48 h after transfection with siRNA markedly inhibited cell invasion in both control and 4E-BP1 knockdown cells, although 4E-BP1 knockdown upregulated Snail expression and promoted cell invasion (Figure 5G, H). Thus, these data suggest that Snail plays a critical role in mediating the effects of mTORC1/4E-BP1 signaling on translational control of cell migration and invasion.

### Targeting translation initiation with 4EGI-1 blocks Snail expression and cancer cell migration and invasion

**Figure 6:**
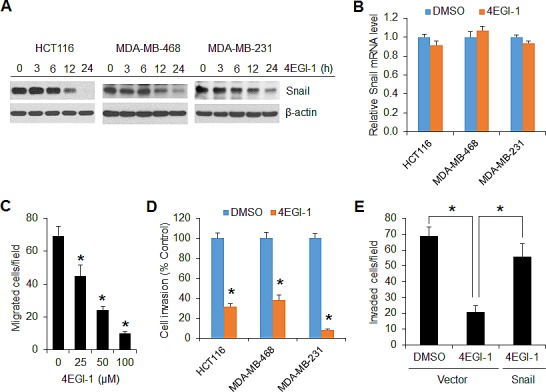
The translation initiation inhibitor 4EGI-1 effectively suppresses Snail expression and migratory and invasive capacities of cancer cells (A) Immunoblot analyses of the indicated cells that were treated with 50 μM 4EGI-1 for the indicated times. (B) Quantitative RT-PCR analysis of mRNA expression of Snail relative to β-actin in the indicated cells that were treated with 50 μM 4EGI-1 or DMSO as control for 24 h (n=3). (C) Migration analysis of HCT116 cells in the presence of 4EGI-1 with the indicated concentrations for 6 h. (D) Invasion analysis of HCT116, MDA-MB-468 and MDA-MB-231 cells in the presence of 50 μM 4EGI-1 or DMSO as control for 30 h, 24 h and 20h, respectively. (E) Invasion analysis of HCT116 cells with expression of vector or Snail in the presence of 50 μM 4EGI-1 or DMSO as control for 30 h. Data shown in graphs represent the mean ± S.E.M. (n=3). * *P* < 0.03.

To further confirm that the cap-dependent translation is required for Snail expression, a selective eIF4E/eIF4G interaction inhibitor, 4EGI-1 that blocks assembly of eIF4F translation initiation complex [31], was tested in HCT116, MDA-MB-468 and MDA-MB-231 cells. As shown in Figure 6A, 4EGI-1 inhibited Snail protein expression in all three cell lines 12 h after drug exposure and profoundly by 24 h. However, the level of Snail mRNA was unaffected by 4EGI-1 (Figure 6B). Similar to the findings obtained from mTORC1 inhibition or repression of cap-dependent translation by 4E-BP1 4A on cell motility (Figures 2 and 3), 4EGI-1 could also inhibit cancer cell migration in a dose dependent manner (Figure 6C). Furthermore, 4EGI-1 markedly inhibited invasive capability of HCT116, MDA-MB-468 and MDA-MB-231 cells (Figure 6D), but overexpression of Snail could largely prevent the inhibitory effects (Figure 6E). These data suggest that exploring a small molecular inhibitor to directly target eIF4F translation initiation complex could represent an alternative strategy for suppression of Snail expression and metastatic potential of cancer.

## DISCUSSION

In this study, we provide substantial evidence to support the notion that the 4E-BP1-regulated cap-dependent translation plays an active role in EMT induction, tumor cell migration and invasion via the selective increased translation of Snail accompanied by its transcriptional repression of E-cadherin expression. Our study demonstrates a distinctive mechanism for the regulation of Snail and its activities in the process of cancer metastasis.

**Figure 7:**
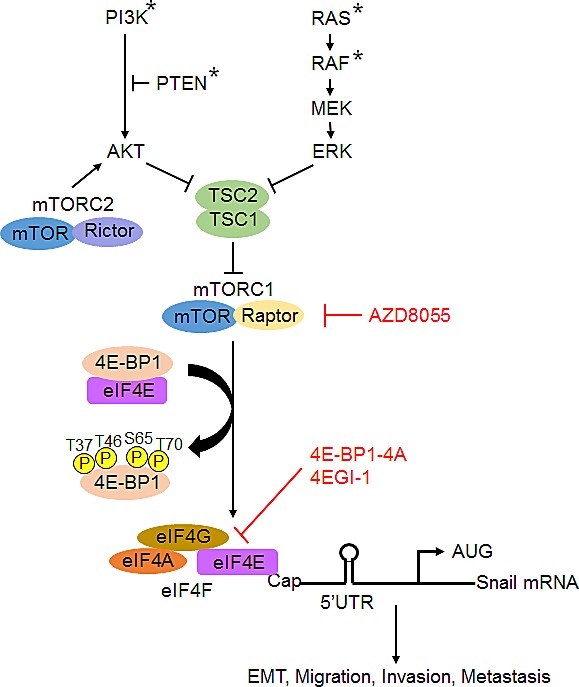
A schematic model for the role of mTROC1/4E-BP1 signaling axis in the translational control of Snail and its biological consequences Arrowheads represent activation; bar-headed lines represent inhibition. * mutation.

Migration and invasion are critical steps in the initial progression of cancer that facilitate metastasis. Emerging evidence shows that Snail-induced EMT via downregulation of E-cadherin is associated with increased migratory and invasive capabilities, and the subsequent systemic spread of cancer cells [14, 15]. The expression of Snail has been shown to correlate significantly with disease relapse and poor prognosis in patients with breast, colorectal and ovarian carcinomas [12-15]. Many studies have demonstrated that Snail is tightly regulated at the transcriptional level by an integrated and complex signaling network including the PI3K/AKT, RAS/RAF/MEK/ERK, RhoGD12, TGFβ/Smad, Notch, Wnt/β-catenin and NFκB pathways [16, 32]. In addition, expression of Snail and its function are also shown to be regulated by posttranslational modifications. GSK-3β, negatively regulated by AKT, has been shown to phosphorylate Snail, leading to its subcellular localization and degradation [17]. Early studies showed that inhibition of PI3K/AKT signaling with the PI3K inhibitors, wortmannin and LY294002, release the suppression of GSK-3β, which leads to subsequent degradation of Snail [17, 33]. However, using the selective AKT inhibitor MK2206 as we and others reported previously [19, 25], we found that inhibition of AKT has no effect on Snail mRNA and protein expression in colon and breast cancer cells (Figure 3B and Supplementary Figure 5). These findings are further confirmed by silencing the AKT upstream regulator, rictor (Figure 3E). Given that wortmannin and LY294002 can also inhibit other PI3K-related enzymes such as mTOR [34, 35], it is thus reasonable to speculate that mTOR may involve in the regulation of Snail expression and its activity. Our data show that Snail, but not other EMT inducers such as Slug and Twist, is specifically downregulated with concomitant increased expression of E-cadherin by the pharmacologic or genetic inhibition of mTORC1, whereas aberrant activation of mTORC1 by TSC2 knockdown exhibits opposite effect. Notably, the decreased expression of Snail by mTORC1 inhibition is not associated with changes in the level of transcription or protein stability. Mechanistically, we identify that the cap-dependent translation repressor 4E-BP1 functions as a key effector of mTROC1 activation on translational control of Snail expression and its subsequent activities as noted by the transcriptional repression of E-cadherin, induction of EMT and promotion of migration and invasion in colon and breast cancer cells (Figure 7). In addition, disruption of cap-dependent translation initiation complex with the selective eIF4E/eIF4G interaction inhibitor 4EGI-1 shows a profound inhibition on Snail expression as well as migratory and invasive abilities of cancer cells. Thus, our findings indicate that Snail activity could be regulated in AKT-independent but mTORC1/4E-BP1-mediated cap-dependent translation initiation mechanism.

A recent report by Evdokimova and colleagues [24] suggested that YB-1 is positively involved in the regulation of Snail expression via cap-independent translation associated with breast cancer aggressiveness, as they showed that inhibition of mTORC1-mediated cap-dependent translation with rapamycin does not affect Snail levels in YB-1-expressing premalignant MCF10AT human mammary epithelial cells. YB-1 is highly expressed in multiple human malignancies including breast and colorectal cancers [36]. Although our data also show that rapamycin has negligible effect on Snail expression and cell invasion in breast and colon cancer cells, we found that the insignificant effect is associated with the weak inhibition of mTORC1-mediated 4E-BP1 phosphorylation by rapamycin. First, we showed that as compared with rapamycin, both mTOR kinase inhibitor AZD8055 and/or specific inhibition of mTORC1 by raptor knockdown exhibit potent inhibition of 4E-BP1 phosphorylation; and both also effectively repress Snail expression and its subsequent activities in colon and breast cancer cell lines with highly expressed YB-1 (Figure 3). Second, the non-phosphorylated mutant 4E-BP1-4A with constitutive inhibition of cap-dependent translation also profoundly inhibits Snail expression and cell motility (Figure 2), as well as colon cancer metastasis, as we reported recently [19]. Third, silencing 4E-BP1 expression significantly rescues translational inhibition of Snail and cell invasion by AZD8055 (Figure 4D, E and Supplementary Figure 4). More importantly, the decreased level of Snail by mTORC1 inhibition is not associated with changes in the level of YB-1 expression (Figure 3A and Supplementary Figure 3). Taken together, these data further support the conclusion that mTORC1/4E-BP1 signaling plays a crucial role in the cap-dependent translational regulation of Snail and its biologic consequences during the metastatic progression of cancer.

Our findings are consistent with recent reports [37-39] showing the role of mTORC1 in the EMT process and translational control of gene expression program for cancer progression. However, a more recent study reported conflicting results. Using normal immortalized human epithelial cell lines and primary epithelial cells, Mikaelian and colleagues [40] showed that genetic and pharmacologic inhibition of mTORC1 triggers EMT associated with upregulation of ZEB1, known to activate EMT, but these effects are not found in cancer-driven cell lines including the MDA-MB-231 breast cancer cell line as we used in this study. Indeed, we found that mTORC1 inhibition represses EMT process in colon and breast cancer cells by specific inhibition of Snail translation attendant with increased expression of the epithelial marker E-cadherin, thus suppressing cancer cell migration and invasion. This discordant effect of mTORC1 inhibition on EMT and cell motility is probably dependent on the mutational status of the cells. Mutations in genes that encode components of the PI3K/AKT and RAS/RAF/MEK/ERK pathways occur at high frequency in cancers including colon and breast cancers [41-43]. We and others have recently shown that mTORC1/4E-BP1 axis largely mediates the effects of the mutational activation of AKT and ERK signaling pathways on translational control of cancer cell motility and metastasis[19] [11, 39]. Thus, it is possible that tumors with mutational activation of AKT and ERK pathways are highly dependent on mTORC1/4E-BP1 signaling for the promotion of EMT, cell motility and metastasis (Figure 7). Our data suggest that targeting mTROC1 with the ATP-site mTOR kinase inhibitors that can effectively activate 4E-BP1 repressive function on cap-dependent translation may provide a promising treatment strategy for blocking metastatic progression of cancer. Many mTOR kinase inhibitors are in early clinical testing. The anti-metastatic effect of these inhibitors and their potential side effect on EMT induction in normal cells should now be evaluated in detail in the clinical studies.

Our results also suggest that directly targeting assembly of eIF4F translation initiation complex (Figure 6) may be an effective alternative to the mTOR kinase inhibitors or combination of AKT and MEK kinase inhibitors as we showed recently [11, 19]. Given the importance of 4E-BP1-regulated cap-dependent translation as a common downstream node that integrates multiple oncogenic signaling pathways for tumor growth and metastasis [11, 19, 44], compounds that mimic 4E-BP1 biochemical function by disruption of eIF4E-eIF4G interaction or target other translation initiation components may be effective for cancer therapeutics. Indeed, several of those translation initiation inhibitors, including eIF4E antisense-oligonucleotides and silvestrol that inhibits the RNA helicase eIF4A, have recently produced encouraging anti-tumor effects with limited toxicity profiles in mouse; and some of these inhibitors have been tested in clinical studies [45-48]. Considering an accumulation of damaging side effects from a combination of therapies that inhibits several canonical signaling pathways impinging upon translation initiation, and the mTOR inhibition-induced feedback activation of upstream receptors and AKT signaling that may reduce anti-tumor effects of mTOR inhibitors [49-52], targeting cap-dependent translation that could simultaneously block upstream oncogenic signals and their downstream targets would hold potential as a future therapeutic strategy against the metastatic progression of cancer.

## MATERIALS AND METHODS

### Cell culture, plasmids, siRNA and shRNA

Human colon and breast cancer cell lines were obtained from the American Type Culture Collection (ATCC, Manassas, VA) and maintained in the appropriate medium with supplements as suggested by ATCC. MK2206, rapamycin and AZD8055 were obtained from Selleck (Houston, TX). The pcDNA3.1-HA-tagged human Snail expression plasmid and the human raptor and rictor shRNA expression plasmids were purchased from Addgene (Cambridge, MA). The specificity of the targeting raptor and rictor sequences has been verified and described previously [26]. The human 4E-BP1 and TSC2 shRNA expression plasmids were from Open Biosystems (Lafayette, CO) and the specificity of the targeting sequences has been verified in our previous study [19]. siRNA pool against human Snail (L-010847) or non-targeting control siRNA pool (D-001810-10) was obtained from Dharmacon (Chicago, IL). For establishing stable transfectants with knockdown of specific protein expression, cell lines were lentivirally infected with the indicated shRNA construct followed by selection with puromycin (2 μg/ml) for one week. HCT116 or HCT116-4E-BP1 knockdown cells with stable expression of HA-tagged 4E-BP1 and 4E-BP1-4A were generated as described previously [11].

### Migration and invasion assays

Migration and invasion assays were performed in Boyden chambers with coated collagen or Matrigel, respectively, as instructed by the manufacturer (BD Biosciences, San Jose, CA) and described previously [19]. Briefly, cells were added to the upper chamber of the transwell insert. Completed medium containing 10% FBS as chemoattractant was added to the bottom chamber. To assess the effect of kinase inhibitors on cell migration and invasion, the inhibitors or their vehicle control DMSO were added to the bottom chamber medium. The plates were incubated at 37°C in 5% CO_2_ for indicated time points. After incubation, cells in the upper compartment were removed with a cotton swab, and cells that migrated or invaded to the filter surface facing the bottom chamber were fixed in 4% paraformaldehyde and stained with 0.2% crystal violet. The numbers of migrated or invaded cells were counted in at least four areas at x 40 magnification using an inverted microscope. All experiments were performed at least twice in triplicate.

### Immunoblot analysis

Cells were lysed in NP-40 lysis buffer as described previously [11]. Equal amounts of total protein were resolved by SDS-PAGE, transferred to membranes, immunoblotted with specific primary and secondary antibodies and detected using chemiluminescence (GE Healthcare Bio-Sciences, Pittsburgh, PA). Antibodies for p-AKT(S473), p-4E-BP1(T37/46), p-4E-BP1(S65), p-4E-BP1(T70), 4E-BP1, eIF4E, Snail, Slug, YB-1, raptor, rictor and TSC2 were from Cell Signaling Technology (Danvers, MA). E-cadherin antibody (67A4) was from Santa Cruz Biotechnology (Dallas, TX). HA antibody was from Novus Biologicals (Littleton, CO) and -actin antibody was from Sigma (St Louis, MO).

### Quantitative RT-PCR

Total cellular RNA was isolated using the RNeasy plus mini kit (Qiagen, Valencia, CA). Equal amounts of RNA were used as templates for all reactions. Double-stranded cDNA was generated by using the SuperScript III First Strand Synthesis System (Life Technologies, Grand Island, NY). Real-time PCR reactions were carried out with specific probes for human Snail (Hs00195591_m1), E-cadherin (Hs01023894_m1) and β-actin (#4352935E) using the StepOne Real-Time PCR system (Applied Biosystems, Foster City, CA).

### Cycloheximide (CHX) chase assay

Cells were treated with CHX (20 μg/ml) and harvested at indicated time points. The cells were lysed in NP-40 lysis buffer and equal amounts of total protein were analyzed by immunoblot. To examine the effect of mTOR inhibition, one set of cells was pretreated with AZD8055 for 30 min before the addition of CHX.

### Cap-Binding Assay

Cell lysates as prepared above were incubated with m^7^GTP sepharose beads (GE Healthcare Bio-Sciences) to capture eIF4E and its binding partners. Precipitates were washed three times with lysis buffer, resuspended in 2x Laemmli sample buffer, and resolved by SDS-PAGE followed by immunoblot with the indicated antibodies.

### Quantification of cap-dependent translation

The 5′-UTR cDNAs of human Snail were obtained by RT-PCR from a HCT116 cDNA library using the primers as described in Supplementary Table 1. These 5′-UTR cDNAs were each inserted immediately upstream from the translation start codon of the renilla luciferase gene in the bicistronic luciferase reporter vector pcDNA3-rLuc-PolioIRES-fLuc, which directs cap-dependent translation of the renilla luciferase gene and cap-independent Polio IRES-mediated translation of the firefly luciferase gene [53]. All sequences were verified by automated sequencing. Cells (80,000) were transfected with each the constructed bicistronic luciferase reporter plasmid (0.2 μg) in 12-well plates using X-tremeGENE Transfection Reagent (Roche Applied Science, Indianapolis, IN). After 24 h transfection, cells were treated with kinase inhibitors for the indicated times, and cell lysates were assayed for renilla luciferase and firefly luciferase activities as described [11]. Cap-dependent renilla activity was normalized against cap-independent firefly activity as the internal control. The renilla/firefly luciferase luminescence ratio was calculated for cap-dependent translational activity.

### Animal studies

Male athymic nude mice (5-6 weeks old) were purchased from Taconic (Hudson, NY, USA) and maintained and treated under specific pathogen-free conditions. The experimental liver metastasis assay was described previously [19]. Briefly, cells with co-expression of firefly luciferase and GFP were injected into the spleen (5×10^6^/mouse) of athymic nude mice (n=6 per group). To monitor metastasis, mice were imaged with luciferase signals using the IVIS Spectrum (Caliper Life Science, Hopkinton, MA, USA) and results were analyzed by Living Image 3.0 software. In addition, the liver metastatic lesions were further examined by GFP imaging.

### Statistical analysis

All experiments were carried out in at least twice. Results are expressed as mean ± S.E.M. where applicable. A two-tailed Student's *t*-test was used to compare the intergroup. Differences between groups were considered statistically significant at *P* < 0.05.

## SUPPLEMENTARY MATERIAL FIGURES AND TABLE


