# Supplementary material for: Loss of 4E-BP1 function induces EMT and promotes cancer cell migration and invasion via cap-dependent translational activation of snail
Source: Oncotarget. 2014 Jun 16;5(15):6015–27. doi: 10.18632/oncotarget.2109 (PMC4171609; doi:10.18632/oncotarget.2109)
Supplement: Supplementary file 1 [file oncotarget-05-6015-s001.pdf]

Loss of 4E-BP1 function induces EMT and promotes cancer cell migration and invasion via cap-dependent translational activation of snail

Supplementary Material

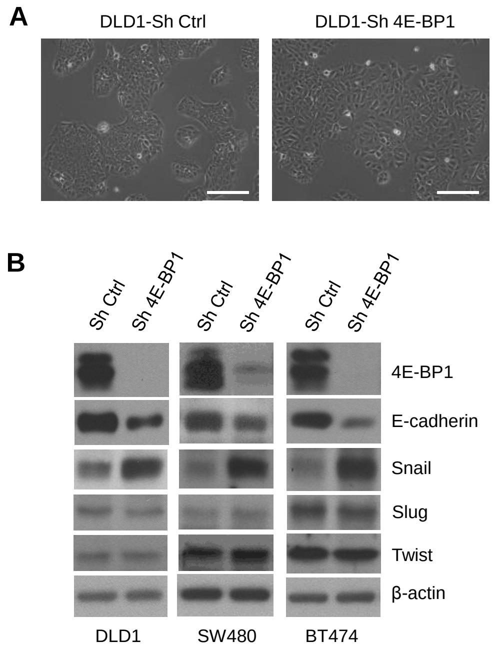

Supplementary Figure 1: Knockdown of 4E-BP1 expression causes cancer epithelial cells to undergo epithelial-mesenchymal transition with selective upregulation of Snail expression. (A) For morphological comparison, DLD1 cells with stable expression of control (Ctrl) shRNA or 4E-BP1 shRNA were photographed using a light microscope. Scale bar = 200 µm. (B) DLD1, SW480 and BT474 cells with stable expression of Ctrl shRNA or 4E-BP1 shRNA were immunoblotted with the indicated antibodies.

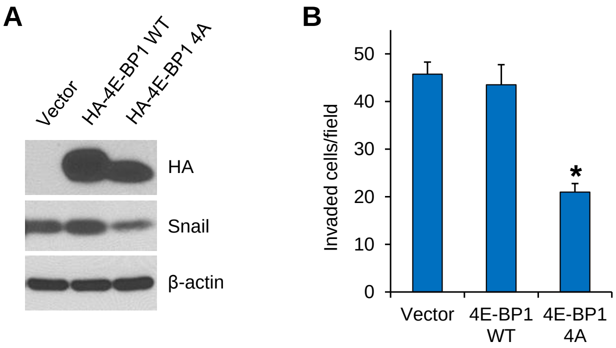

Supplementary Figure 2: Expression of a dominant active 4E-BP1 mutant inhibits Snail expression and invasive ability of MDA-157 breast cancer cells. (A) Immunoblot analysis of MDA-157 cells with stable expression of vector, HA-4E-BP1 WT or HA-4E-BP1 4A. (B) Transwell invasion analysis of MDA-157 cells with stable expression of vector, HA-4E-BP1 WT or HA-4E-BP1 4A over 30 h. The results represent the mean number of invaded cells per field ± S.E.M. (n=3). * $P < 0.02$ for 4E-BP1 4A versus 4E-BP1 WT or vector.

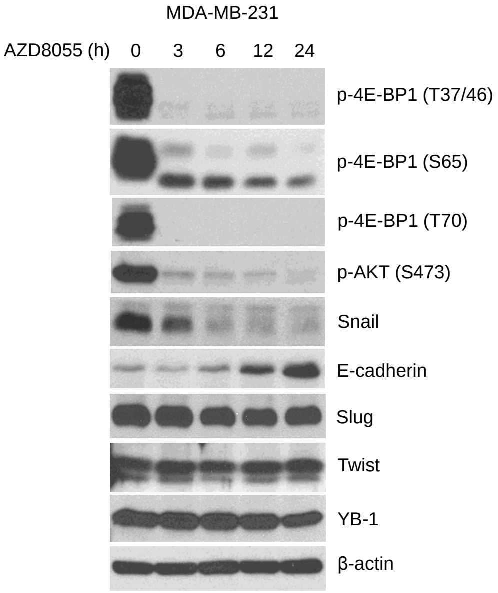

Supplementary Figure 3: AZD8055 selectively inhibits Snail expression followed by induction of E-cadherin in MDA-MB-231 breast cancer cells. MDA-MB-231 cells were treated with 500 nM AZD8055 for the indicated times. Cell lysates were immunoblotted with the indicated antibodies.

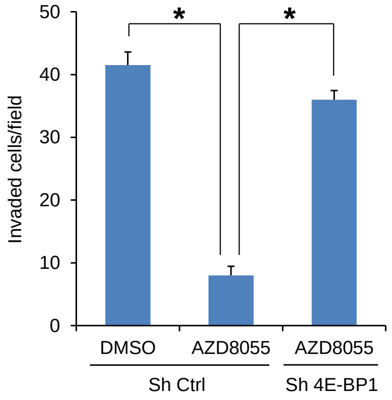

Supplementary Figure 4: Silencing 4E-BP1 expression markedly prevents the inhibitory effect of AZD8055 on cell invasion. Invasion analysis of HCT116 cells with stable expression of control shRNA or 4E-BP1 shRNA in the presence of 500 nM AZD8055 or DMSO as control for 30 h. The results represent the mean number of invaded cells per field ± S.E.M. (n=3). * $P < 0.02$.

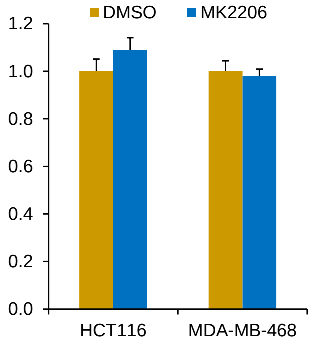

Supplementary Figure 5: AKT inhibition has no effect on Snail mRNA expression. Quantitative RT-PCR analysis of mRNA expression of Snail relative to β-actin in the indicated cells that were treated with 1 μM MK2206 or DMSO as control for 12 h (n=3).

Supplementary Table 1: The primers used to generate 5’-UTR (underlined) of Snail.

5'-UTR	Forward Primer Sequence	Reverse Primer Sequence
Snail	TCTTTGCTAGC<u>ATTCATTGCGCCGCGGCAC</u>	TTGTTGCTAGC<u>AGTGGTCGAGGCACTGGGGT</u>
